# Comparing Comprehension of a Long Text Read in Print Book and on Kindle: Where in the Text and When in the Story?

**DOI:** 10.3389/fpsyg.2019.00038

**Published:** 2019-02-15

**Authors:** Anne Mangen, Gérard Olivier, Jean-Luc Velay

**Affiliations:** ^1^Norwegian Reading Centre, University of Stavanger, Stavanger, Norway; ^2^Laboratoire Interdisciplinaire Récits Cultures Et Sociétés (LIRCES EA 3159), Université Nice Sophia Antipolis, Nice, France; ^3^Laboratoire de Neurosciences Cognitives (UMR 7192), CNRS and Aix-Marseille Université, Marseille, France

**Keywords:** reading comprehension, kinesthetic feedback, cognitive map, print-book, kindle, long text reading

## Abstract

Digital reading devices such as Kindle differ from paper books with respect to the kinesthetic and tactile feedback provided to the reader, but the role of these features in reading is rarely studied empirically. This experiment compares reading of a long text on Kindle DX and in print. Fifty participants (24 years old) read a 28 page (∼1 h reading time) long mystery story on Kindle or in a print pocket book and completed several tests measuring various levels of reading comprehension: engagement, recall, capacities to locate events in the text and reconstructing the plot of the story. Results showed that on most tests subjects performed identically whatever the reading medium. However, on measures related to chronology and temporality, those who had read in the print pocket book, performed better than those who had read on a Kindle. It is concluded that, basically comprehension was similar with both media, but, because kinesthetic feedback is less informative with a Kindle, readers were not as efficient to locate events in the space of the text and hence in the temporality of the story. We suggest that, to get a correct spatial representation of the text and consequently a coherent temporal organization of the story, readers would be reliant on the sensorimotor cues which are afforded by the manipulation of the book.

## Introduction

### The Digitization of Literary Reading

Overall, in the western world, reading is increasingly digitized. Due to the popularity of handheld, portable digital devices such as e-readers (e.g., Kindle) and tablets (e.g., iPad), also long-form literary reading is becoming screen- rather than print-bound. This transition invites a number of research questions pertaining to the role of substrate affordances (e.g., screen displays and paper) on cognitive and emotional aspects of narrative, literary reading.

In striking ways, the move from paper to screen makes evident that reading is a case of human-technology interaction (Mangen and van der Weel, 2016). In addition to more commonly addressed perceptual and cognitive components of discourse processing, reading typically entails manual engagement with a device (e.g., a print pocket book, an e-reader or a tablet). Different devices have different user interfaces and material affordances (Gibson, 1977), and the substrate of paper in a print book provides sensorimotor contingencies (O’Regan and Noë, 2001) that differ from those of texts displayed on a screen. Print texts are physically and tangibly contiguous with the medium, whereas digitized texts are physically separable from their medium. This enables a digital device to store a large number of texts and other content.

However, we know little about the ways in which such seemingly subtle differences may interact with cognitive and experiential aspects of reading. Reading scholars of a theoretical ilk have emphasized how reading is more multisensory than commonly acknowledged: “Smell and sight are relevant senses when it comes to reading [,]” says Naomi Baron, “but touch may well be the most important” (Baron, 2015, p. 142). Analogously, Mc Laughlin notes how “the feel of the book to the hand, the smell of the paper, the haptic pleasure of manipulating the screen […] reinforce and deepen the habit of reading” (Mc Laughlin, 2015, p. 31). Broadly conceptualized, “haptic” (from Greek haptikos = able to touch) refers to the sense of touch. As such, it encompasses both “passive” (cutaneous [tactile]) and “active” (proprioceptive; kinesthetic) sensory processes. In the research literature, terms such as haptic, force feedback, and kinesthetic are often used interchangeably. In this article, *kinesthetics* will refer to the combined (passive) sense of touch (e.g., pressure; temperature) and the (active) aspects entailed in proprioception (the sense of the relative position of muscles, joints and tendons) and kinesthesia (the sense of movement).^1^ Questions concerning the role of haptics and kinesthetics in reading rise to prominence with the current digitization, and the increasing use of e-readers and tablets is an occasion to put such theoretical assumptions to empirical scrutiny.

### Reading on Paper and Screens

During the past couple of decades, scientists and scholars in reading research have increasingly taken an interest in potential effects of technological interfaces on aspects of reading and learning, more generally. A large number of empirical studies have been carried out, comparing reading on computer screens and, more recently, on tablets and smartphones, with reading on paper (see Baron, 2015 for an overview). This research spans a range of disciplines and a variety of methodologies, assessing the effects of screen properties on, e.g., perceptual processes (Roschke and Radach, 2016), memory and recall (Morineau et al., 2005; Kerr and Symons, 2006; Porion et al., 2016), comprehension (Mangen et al., 2013; Margolin et al., 2013; Rockinson-Szapkiw et al., 2013; Hermena et al., 2017; Hou et al., 2017; Xu et al., 2017; Salmerón et al., 2018) and metacognition/calibration (Ackerman and Goldsmith, 2011; Norman and Furnes, 2016; Sidi et al., 2016, 2017). More recently, research has begun to address topics such as ergonomics (Köpper et al., 2016), issues of medium materiality (Hou et al., 2017) and interactions between medium and particular text types/genres (Rasmusson, 2014; Singer and Alexander, 2017a). As for effects of medium on reading comprehension, the issue remains somewhat unsettled (see Hermena et al., 2017; Xu et al., 2017). Some empirical studies have found reading comprehension to be superior on paper (Kim and Kim, 2013; Mangen et al., 2013; Rasmusson, 2014), whereas others show no differences between paper and screen (Margolin et al., 2013; Rockinson-Szapkiw et al., 2013; Porion et al., 2016). However, a recent meta-analysis (Delgado et al., 2018) of 54 experiments published between 2000 and 2017 comparing the reading of comparable texts on paper and screens does find an advantage for paper both for between-participants and for within-participants studies. The meta-analysis revealed three significant moderators for this main finding: (i) time frame (i.e., the advantage for paper-based reading was stronger in time-constrained reading than in self-paced reading); (ii) text genre: the paper-based reading advantage was consistent across studies using informational text or a mix of informational and narrative texts, but there was no difference for narrative-only texts; and (iii) publication year: contrary to assumptions of “digital natives” becoming better screen readers with increasing screen exposure and experience, the meta-analysis found that the advantage of paper-based reading in fact increased from 2000 to 2017 (Delgado et al., 2018).

In a similar vein, a systematic literature review of empirical research (Singer and Alexander, 2017b) found that when participants were reading texts for depth of understanding and not solely for gist, print was the more effective processing medium. Moreover, with respect to reader preferences and habits, a recent large international survey (Mizrachi et al., 2018) with more than 10,000 participants found that, for academic reading, a broad majority reported a preference for print, especially when reading longer texts. Interestingly, participants reported that they felt they remembered the material better and were better able to focus when reading in print, compared to when reading digitally (Mizrachi et al., 2018).

On another note, some studies have revealed a discrepancy between objective and subjective measures. A study (Kretzschmar et al., 2013) combining EEG, eye tracking and questionnaires found that participants overwhelmingly preferred paper over digital reading, but comprehension accuracy did not differ between media.

### Visual and Ergonomic Affordances of Paper and Screen Substrates

Screen technologies vary with respect to visual ergonomics. Laptop/computer and tablet (LCD) screens emit light and hence are found to cause eyestrain and visual fatigue (Baccino, 2004; Blehm et al., 2005; Yan et al., 2008). In contrast, e-readers (e.g., Kindle) are based on electronic ink, a screen substrate specially designed to mimic paper (Siegenthaler et al., 2011). Due to a stable image, wider viewing angle, and the fact that they merely reflect ambient light rather than emitting light, e-readers are more reader friendly than tablets and computers, particularly for longer texts. A growing body of evidence indicates that the readability of e-readers is experienced as being equal to, and occasionally better than, that of paper (Siegenthaler et al., 2011, 2012; Benedetto et al., 2013). In addition, with screens it is possible to scroll up and down the pages of a book. However, scrolling is known to impede readers’ capacity to create an effective mental map of the text (Hou et al., 2017). For these reasons, and unlike earlier studies on narrative reading on paper and screen (e.g., Mangen and Kuiken, 2014; Singer and Alexander, 2017a), we used a Kindle in the present study.

However, when reading a long text included in a book, there is more to reading than meets the eye. Indeed, for a long text printed on many pages, reading does not only involve the eyes: it also involves the hands. Whereas a text displayed on a Kindle and in a print book may be similar with respect to visual properties (the texts look identical on paper and on screen), the two texts differ with respect to the ergonomic affordances of the substrate. Manipulating a printed-book and an e-book is not the same. When reading print text on paper, readers have immediate sensory – kinesthetic and tactile – access to text sequence, as well as to the entirety of the text. The sensorimotor contingencies of paper gives book readers visual as well as kinesthetic feedback to their progress through a text (Mangen and Kuiken, 2014). To know where they are in a text printed on paper, readers have at their disposal several cues: they can have a look at the page number (visual cue), but they can also refer to tactile-kinesthetic cues given by the handling movements informing about the repartition of the weight of the pages on the left and on the right of the current page, and consequently on the number of pages already read and on the number of pages still to read. In addition, the page turning movements might also somehow inform about the number of pages already read. Conversely, screen readers have only visual information on progress and spatial location (e.g., by page numbers or progress bars).

During holding, manipulation of the objects allows to gather information about them even without the aid of vision (Hatwell et al., 2003; Ittyerah, 2017). Thanks to manipulation movements, we build an internal representation of the spatial characteristics of the objects. Print books are special objects whose size, weight and volume are a direct indication of the length of the text. This is not the case when reading e-books.

Now, it is often reported by digital readers that they feel it difficult to have a clear representation on the entirety of the text and to localize a given part of information within the text (e.g., Rose, 2011), and there is some empirical evidence supporting this phenomenon (Mangen and Kuiken, 2014). For this reason, readers of long documents on computer screen often prefer to print the document (Baron et al., 2017; Mizrachi et al., 2018). For a reader, being able to situate where he/she read a given piece of information in the text is important because the relative position of events presented in the space of the text is related to the moment these events took place in the time of the story. For certain types of texts, such as texts relying on plot (the unfolding of the story in a clear logical and temporal fashion), a clear representation of the temporal relationships between the events in a story is crucial to build a coherent situation model sustaining the comprehension of a text. Temporal links between events are generally equivalent to causal connections between these events (usually causes come before their consequences) and causal links between events is one of the components of the situation model (Kintsch and van Dijk, 1978; Kintsch, 1998).

When reading on a digital device, haptic and kinesthetic cues such as these are not available to the reader. When reading on a Kindle, for instance, the reader has access to visual cues only with respect to the spatial location of text segments, and to the temporal progression of reading. Therefore, the main hypothesis of this study was that reading a relatively long, linear text on a Kindle generates difficulties to localize relevant events within the space of the text and within the time of the story.

Still, reading experiments using long narrative texts as stimuli is scarce. In what may have been the first experiment to compare narrative engagement when reading a “real,” somewhat longer (ca. 2700 words) narrative text on iPad and on paper, Mangen and Kuiken (2014) found that the paper group reported a better grasp of text length and of their location in the text than the iPad group. Interestingly, however, they found no correlation between this “sense of dislocation” with readers’ reported sense of narrative engagement, nor did the groups differ on cognitive measures (Mangen and Kuiken, 2014).

The present study elaborates Mangen and Kuiken’s study by (i) using a Kindle DX instead of an iPad; (ii) using a longer, literary text in its entirety; and (iii) focusing on potential effects of the Kindle’s lack of, specifically, tactile feedback on spatial location and progress. In addition, in the present study the stimulus text in both conditions is matched for surface dimensions. Whereas Mangen and Kuiken (2014) opted for using the Kindle app for iPad to ensure comparable reader friendliness across conditions, we modeled the print stimulus on the surface measures of the Kindle, so that page layout, margin sizes, sentence number and length, and number of pages were identical in Kindle and in print. This matching was done in order to avoid visual discrepancies as a potential confound, and was important in light of our attempt at disentangling potential effects due to visual ergonomics on the one hand, and effects due to haptics and kinesthetics on the other. We combined cognitive measures of recall and comprehension with subjective measures assessing experiential aspects of reading a mystery short story on Kindle and in a print pocket book. Specifically, we combined word- and sentence recognition tasks, factual recall measures and assessment of readers’ ability to reconstruct spatial and temporal aspects of the text with rating scales assessing aspects of readers’ engagement.

## Materials and Methods

### Participants

Fifty young adults (mean age 24 ± 3.9; 32 females) participated in the experiment. All participants had normal or corrected to normal vision. They signed a written and informed consent after the procedure was fully explained and were paid for participation. Two participants with learning difficulties were discarded prior to the experiment and replaced by two new subjects. Prior to the reading session, participants completed a questionnaire asking about their study level, reading habits, and familiarity with e-readers. Upon asking participants about their experience with Kindle (or similar device) reading, it was found that some were casual users of e-books. Only two participants among 50 were expert Kindle readers who did all their reading, including literary reading, on their own Kindle. Groups were matched at best with respect to demographic variables (age, gender, education) and reading habits (reading frequency). Considering all these criteria, these two participants were assigned to the Kindle group. Therefore, they read on their preferred device but without unbalancing the two groups regarding e-reader familiarity (see Table 1). After the reading session, we checked with the participants if they had read the story before. This was not the case for any of them. The study had prior approval by the Ethics Committee of the Aix-Marseille University (N° RCB 2010-A00155-34) and the CNRS. Participants signed a written informed consent form prior to the study. They were fully debriefed following their participation.

**Table 1:** Descriptive statistics: demographics and reading habits.

Medium	Sample	Age	Number of years at university	Reading frequency^a^	E-reader familiarity^b^
Print	*N* = 25 (16 females); 4 left-handed	23.6 ± 3.8 years	4.2 ± 2.0	2.6 ± 1.0	0.3 ± 0.7
Kindle	*N* = 25 (16 females); 5 left-handed	23.8 ± 4.1 years	4.2 ± 2.1	2.4 ± 1.2	0.4 ± 0.9


*^*a*^Number of books read per year: 0–3 books = 1; 3–5 books = 2; 5–10 books = 3; >10 books = 4. ^*b*^E-reader familiarity: have never used a Kindle or similar device = 0, have occasionally used a Kindle or similar device = 1, have often used a Kindle or similar device = 2, always using a Kindle or similar device = 3.*

### Materials

#### Stimulus

The stimulus was a 28-page (about 10,800 words) mystery story by Elizabeth George, titled *Lusting for Jenny, Inverted*. The text appears in a collection of short stories (George, 2010). *Lusting for Jenny, Inverted* is a quite conventional mystery story, a “clever tale of lust, greed and false pretenses” (Goodstein, 2010). It tells the story of an older woman, Jenny, who is called to be the executrix of her aunt’s will. Jenny feels unfulfilled with her comfortable but boring housewife life in Long Beach, California. When she he comes to the isolated Washington state island community to settle her aunt’s estate, she meets a charming young man who seems to offer her romance and excitement. They embark on an affair that seems to promise complete fulfillment of all of Jenny’s desires, but things get very complicated when a very valuable stamp collection is discovered as part of the estate. The story is plot-based, easy to read and progresses in a linear fashion, without any significant analepses (flashbacks) or prolepses (foreshadowing) (Genette, 1983).

#### Media Dimensions (Print Book and Kindle)

**FIGURE 1:**
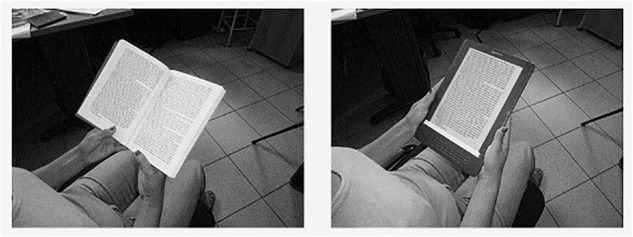
The print pocket book and the Kindle. The left-hand page in the print book corresponds to the page displayed on the Kindle.

For the print book condition, the 28 pages of the text appeared in a 250-page long dummy pocket book (see Figure 1). Ten blank pages preceded the first page of the story, and all pages following the end of the story, were blank. The text was printed recto-verso, just like in a “real” book. The pocketbook was 20.0 cm in height, 14.0 cm in width and 1.8 cm thick. Its weight was 328 g. Great methodological care was taken to ensure similarity of the visual ergonomics of both reading display. The same pdf file was used to create both the print and the e-book. The surface dimensions of each page (font size, sentence length, size of line spacing and margins, letters size) were defined to match exactly those of the screen of the Kindle. In addition, the electronic ink technology used in the Kindle allows long-form reading without visual fatigue which could have a detrimental effect on reading.

The Kindle was a Kindle DX, measuring 26.5 cm in height, 18.0 cm in width and 0.5 cm thick. The weight was 540 g. The screen dimensions were: 20.0 cm × 14.0 cm (see Figure 1). The reader turned the page by clicking on two buttons on the right side, marked by color-codes and “forward” and “back” labels. In order to ensure maximum comparability with the print book, all other Kindle affordances were disabled (e.g., the keyboard; search options; bookmarking). Before reading, the participant was briefly shown how to turn the pages.

We were particularly interested in potential changes in the participants’ ability to locate events in the text. To avoid that the participants referred to the page numbers to see how many pages they had read, we stripped the texts in both conditions for page numbering and we concealed the progress bar of the Kindle.

### Tasks and Procedure

Participants were explained that they participated to an experiment comparing paper and e-book reading and that they have been assigned to one of the reading groups. They were not informed of the exact purpose of the experiment, but only that they will have to read a short story and that they will be asked to answer some questions after their reading. They were not told about the content of the questions. The session took place in a quiet room, and the participant sat in a comfortable chair equipped with armrests. The experimenter was seated in the opposite corner of the room, facing away from the participant. Participants were handed the book opened on the first page and asked to start reading. When the participants had finished reading, the experimenter registered the actual reading time and the participants were asked to estimate the duration of their reading (number of minutes). Although it is not a common assessment in reading experiments, we used the estimated reading time as an indirect index of how far the readers were transported in the story: the longer the estimated time, the lesser the transportation of the reader and vice-versa. Then, the participants completed the tests in the following order:

- *Transportation and Engagement Scale*: a shortened, 33-item measure assessing aspects of readers’ sense of transportation, narrative engagement and resistance to distraction, largely adapted from Busselle and Bilandzic’s Narrative Engagement Scale (Busselle and Bilandzic, 2009)^2^. This scale has been used extensively in experiments assessing readers’ emotional engagement in narrative fictions (see e.g., Kuijpers et al., 2014).

Assessments of readers’ comprehension were inspired by Van Dijk and Kintsch’s (1983) model of comprehension, defining comprehension as an outcome of the interaction of features of the text and the readers’ knowledge. Van Dijk and Kintsch’s (1983) model distinguishes between comprehension at text base level (corresponding to the propositional representation of the text at micro- and macro-levels), and the situation model (referring to the representation of the text which is integrated with readers’ prior knowledge), accommodating a nuanced assessment of readers’ mental representations of different textual features at several levels In the present experiment, short-term recall, text-based (surface) level representation were assessed by recognition tasks, whereas situation model representation was assessed with measures tapping into readers’ reconstruction of the story Short term memory of words and sentences denotes the attention readers paid to the text during reading and the text comprehension.

- The *Word Recognition Task* consisted of 90 words. Participants were asked “Was this word present in the text you just read?” on a computer screen and the response was given using the arrow keys of the keyboard.
- The *Sentence Recognition Task* contained 40 sentences. Participants were asked “Was this sentence present in the texts you just read?” with the procedure being same as for the word recognition test.

Participants’ factual recall was assessed with a *Content Recall Questionnaire* comprising 64 multiple-choice items in five categories: (i) *Characters*: 23 questions about the story characters, their physical characteristics, personality features, relationships between characters (sample item: “How old was Jenny when she had her first child?”); (ii) *geographical setting*: 9 questions about the locations of the story, assessing readers’ recollection of spatial content (sample item: “What is the name of the island where the story takes place?”); (iii) *key locations*: 9 questions about key locations in the story (sample item: “In which room in the cottage was Marion Mance found dead?”); (iv): *objects*: 6 questions about key objects in the story (sample item: “What is the estimated value of the ‘inverted Jenny’ stamp?”); and (v) *time and temporality*: 7 questions assessing readers’ recollection of temporal dimensions of the story, e.g., time lapse between events, chronology and duration of events (sample item: “For how long do Ian and Jenny stay at Blackberry point before the owners come back?”). Participants gave their response orally, and the examiner registered the response.

- “*Where in the text?”*: in a measure inspired by the Rothkopf (1971). Experiment we asked participants to locate 16 sentence-length condensations of key events to their correct place in the text: the first (pages 1–9), second (pages 10–18), or third part (pages 19–28) (sample item: “When did Ian discover the value of the ‘Inverted Jenny’ stamp?”). The question format sentences were presented, one-by-one, on the screen and the participant gave her response orally. The examiner registered the response.
- *Plot Reconstruction Task*: 14 sentence-length condensations of key events of the story were written on laminated pieces of paper and were presented in a shuffled order to the participant. Participants were asked to sort them in the correct order, in accordance with the plot. Upon completion of the task, the resulting order was registered by the experimenter.

### Statistical Analysis

In all tests, data from both groups were compared using independent samples *t*-tests, except for the factual recall questionnaire and the ‘where in the text?’ test for which the data were submitted to a two-way ANOVA with repeated measures.

## Results

### Objective and Subjective Measures of Reading Time

Results are presented in Table 2. There was no difference between reading media with respect to objective reading time [58 min in average, corresponding to a reading speed of 186 words per minute (wpm), *t*(48) = 0.34, ns], and reading time estimates were nearly identical across groups [50 min, *t*(48) = 0.06, ns].

**Table 2:** Mean (SD) actual and estimated reading times with both reading medium.

Medium	Actual reading time	Estimated reading time
Print	59 (17)	50 (25)
Kindle	57 (20)	50 (18)


### Transportation and Engagement Scale

For each participant, responses were summarized for all 33 items of the scale. Results showed no significant between-group difference between ‘print’ and ‘kindle’ groups scores [140 and 149 respectively; *t*(48) = 0.2, ns].

### Word Recognition Task

The mean number of correct responses in this test was 59.8 (±7.5) and 61.2 (±6.9) with the print book and kindle respectively. The difference was not significant [*t*(48) = 0.70, ns].

### Sentence Recognition Task

The mean number of correct responses in this test was 27.5 (±4.4) and 26.5 (±4.6) with the print book and kindle respectively. The difference was not significant [*t*(48) = 0.76, ns].

### Factual Recall Questionnaire

Results are presented in Table 3. As the number of questions differed across sentences categories, we calculated the percentage of correct responses in each category by dividing the number of correct responses by the number of questions in the category. Then, the percentages were arc sinus transformed to be analyzed by means of a two-way ANOVA with category as a within-subject factor and reading medium (print vs. Kindle) as between-subjects factor. The mean number of correct responses was 63.5 and 60.5% for print and e-book respectively [*F*(1,48) < 1, ns]. The number of correct responses differed as a function of question category [*F*(4,192) = 13.2, *p* < 0.001, η^2^ = 0.22]. Because we were particularly interested in the “time and temporality” questions we made a specific planned comparison between the two reading media in this category which revealed a statistically significant difference [*F*(1,48) = 4.1, *p* < 0.05, η^2^ = 0.08].

**Table 3:** Factual Recall Questionnaire: Rate of correct responses (%).

Reading medium	Characters	Geographical setting	Key locations	Objects	Time and temporality
Print	76.4	60.0	62.5	61.6	57.1
Kindle	72.2	59.4	57.1	69.6	44.0


### ‘Where in the Text?’ Measure

**FIGURE 2:**
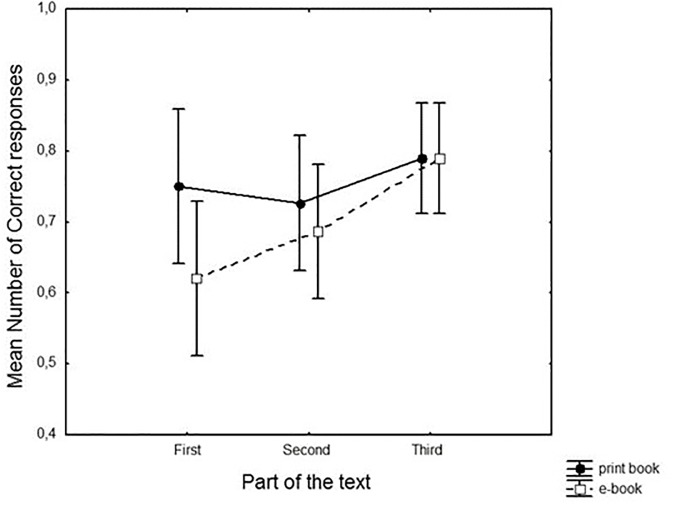
Where in the text: rate of correct responses (%).

There was no significant difference between the two reading media [*F*(1,48) = 1.91, ns]. The ‘part of the text’ factor was close to significant [*F*(2,96) = 2.97, *p* < 0.057]. Indicative of a well-known recency effect (Murdock, 1962; Gershberg and Shimamura, 1994), participants scored better for questions concerning the last third of the text, compared to the first and second part (Figure 2). Although this effect may seem larger in the Kindle group, the ‘medium’ by ‘part of text’ interaction was not significant [*F*(2,96) = 1.1, ns). However, the medium comparison for the first part only revealed a significant effect (*p* < 0.05, η^2^ = 0.06). In other words, the print book readers gave more correct responses than the Kindle readers for questions concerning the first part of the text.

### Plot Reconstruction Task

**FIGURE 3:**
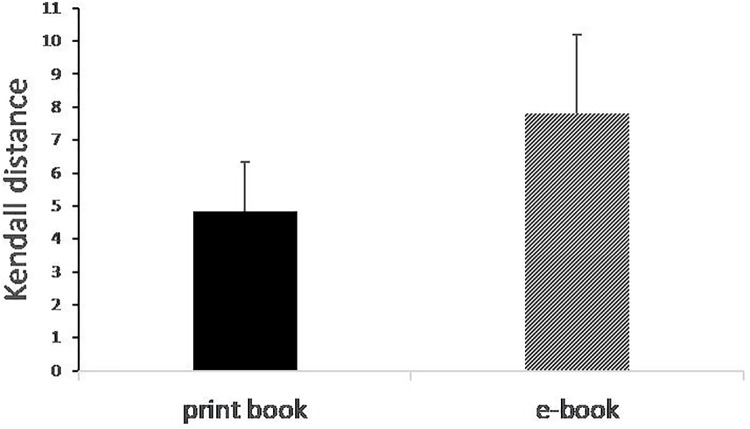
Plot reconstruction task: distance from correct order.

To measure the distance between the correct arrangement of events according to the plot, and the arrangement proposed by the participant, we used the Kendall’s tau rank distance (Kendall, 1938, 1962), a statistical measure that corresponds to the number of pairwise disagreements between two ranking lists. The more the ranking list given by the participant is far from the exact list, the larger the distance Kendall is^3^. The mean distance was 4.8 for the ‘print’ group and 7.8 for the ‘Kindle’ group, and a *t*-test showed that the between-group difference was statistically significant [*t*(48) = 2.03, *p* < 0.05; η^2^ = 0.08], meaning that the print group performed better (with a shorter distance from the correct order) than the Kindle group on this measure (Figure 3).

### Correlation Between ‘Where in the Text?’ and Plot Reconstruction Tests

Because both tests were supposed to assess the capacity to localize events in the space of the text and to replace events of the story in the correct order, we supposed that the performance in both tests would be somehow linked. Therefore, we made a regression analysis of the rate of correct responses in the ‘where in the text?’ test and the Kendall distance in the ‘plot reconstruction test across all subjects (both reading media confounded). This analysis revealed a significant correlation between both variables [*R* = -0.356, *F*_(1,48)_ = 6.98, *p* < 0.02]. The correlation was negative, therefore the greater the number of correct responses given in the ‘where in text?’ test, the smaller the distance between the exact order of the ranking list and the list reconstructed by the participant.

## Discussion

The main objective of this study was to assess the effect of material affordances of a Kindle on cognitive aspects of narrative reading. More specifically, we tested whether the Kindle’s lack of kinesthetic and tactile feedback on the distribution and location of text elements may negatively affect aspects of readers’ cognitive reconstruction of a narrative reading, in particular, with respect to its temporal and chronological dimension.

The question of the material affordances of the reading support has never been really explored and in order to address this question specifically, we made some methodological choices, the most important being the length of the text to read. Obviously, if the kinesthetic feedback generated by the book manipulation matters, it can be only during long-form reading. Therefore, in this experiment we decided to have adult readers to read a long text (10,800 words), requiring approximately 1 h reading and hence a substantial manipulation of the book. Such a long reading time is beyond those usually required in experiments devoted to reading comprehension. Comprehension of long texts involves short- and long-term memory of the text and building a coherent situation model representation, a major feature of which is its global organization into main points and subordinate points (Kintsch, 1998). This situation model might depend partly on a cognitive map, a spatial representation, of the text (Payne and Reader, 2006; Li et al., 2013; Hou et al., 2017) that the readers automatically build during reading and which might be less precise when reading an e-book as compared to a print book.

The results showed that, on most of the measures, there were no differences between the Kindle and the print pocket book. This is in line with some recent reviews of reading comprehension on paper and screen (Hermena et al., 2017; Xu et al., 2017). This was particularly the case concerning the reading time which has been a matter of controversies in the literature, with some authors reporting a slower reading with tablets and others no difference. In the present study, the reading time did not differ according to the type of reading support. Beside the actual reading time, the level of engagement of the reader in the reading was assessed by a questionnaire, and more indirectly, by the subjective reading time. Neither of these measures yielded differences between the reading media, thus we may assume that readers’ emotional engagement were roughly the same with both types of books. Furthermore, readers’ score on the word- and sentence-recognition tests did not differ in the two conditions, suggesting that surface reading and attention paid to the text did not differ between the print book and the e-book. Finally, in the recall questionnaire, most of the questions about the text content did not yield any differences. To conclude, most of the measures we used to assess the text comprehension did not show any differences between print- and e-book.

Nevertheless, some differences were observed between the media regarding tasks tapping into readers’ ability to correctly reconstruct temporal and chronological aspects of the text. In the recall questionnaire, on measures related to time and temporality, those who had read in the print pocket book, performed better than those who had read on a Kindle. The ‘where in the text?’ test, which was specifically devoted to assessing the capacity of the readers to localize the events in the text, also yielded results going in the same direction: paper readers were better at localizing the events than the Kindle readers when the events were the furthest from the end of the book (or at the beginning of the story). Hence, the mental representation of the part of the text corresponding to the reading events which were the most remote in time (at the time of the task), was stronger for those who had read on paper than for those who had read on Kindle. Finally, the plot reconstruction test, which directly assessed the mental representation of the chronology of the story, indicated that print book readers had a more coherent situation model than e-book readers.

How may these differences between the two reading supports be interpreted? First, it is worth emphasizing here that memory of the text *per se* was not affected by medium. The word and sentence recognition tests and the majority of the recall questions yielded the same results in both reading media. Therefore, the differences on some of the measures cannot be related to differences in memory in the two media, nor can they be explained by differences in attention paid to the text during reading. If either of these had been the case, one would have expected the Kindle group to have performed differently on all the tests.

We suggest that these differences could be interpreted as an indication that the sensorimotor assessment of the device may be related to certain aspects of cognitive processing and, moreover, that these aspects are specifically related to reading longer linear texts. The text used in the present experiment was one in which the temporal unfolding of events in the story corresponded closely with their spatial localization in the text (e.g., no major flashbacks) so that there was a correspondence between “where in the text” and “when in the story” events occur. This is shown by the significant correlation observed between both tests results. In other words, the better the readers were able to locate events in the space of the text, the better their representation of the chronology of the story was. In this respect, the fixity of a text presented on the physical substrate of paper provides material placeholders, functioning to off-load cognitive processes during reading. Such off-loading may be of particular importance when reading certain kinds of texts – for instance, long narrative texts in which the distribution of elements (e.g., story events and characters interactions) according to the unfolding of a narrative (i.e., the plot) matters. On the other hand, the intangibility of a text on a Kindle and lack of fixed cues – “material anchors” (Schilhab, 2017) – to length and spatiotemporal extension of the text may also contribute to a loss of orientation with respect to readers’ assessment of the temporal relations between events in the text. The lack of fixity (and hence less informative tactile feedback) of the text displayed on the Kindle may have left readers less confident about where they are in the text corpus (volume), and this lack of confidence may have had a negative effect on their ability to build a correct representation of the story. Of related relevance, research has shown that having a good mental representation of the spatial representation or layout of the text supports reading comprehension (Baccino and Pynte, 1994; Cataldo and Oakhill, 2000; Hou et al., 2017). Somehow, the material anchors of paper seem to have provided better scaffolding for aspects of the mental reconstruction than the e-ink display of the Kindle. However, any conclusive interpretation of these results is challenged by the fact that establishing causality is linked to the processing of order events, hence, inferior ordering of events could have been expected to negatively affected readers’ mental construction of causality, in turn resulting in poorer overall comprehension. This was not the case in the present experiment, as readers in both conditions performed equally well on the comprehension measures. Instead, the differences observed may be more closely related to the participants’ ability to *correctly locate single events in time*, rather than their ability to reconstruct the order of events *per se*, on a global level. Future research should be designed to enable more precise assessments of the ways in which the affordances of reading substrates – screen displays and paper – may differently affect distinct, but closely related, aspects of mental reconstruction of chronology and temporality during perhaps especially long-form reading. In this task, developing improved measures for inter-events associations is pivotal.

Hou et al. (2017) distinguished two mechanisms to explain why reading on a digital support versus on paper might result in different reading outcomes. The first mechanism contends that, because they lack fixed visual anchors, screens make it difficult for readers to construct an effective spatial representation of the text and, in turn, readers are impaired in their capacity to locate pieces of information in text. The second mechanism they evoked is concerned with the sensorimotor engagement with the paper or digital texts, which was highlighted in the present experiment. We think that these two mechanisms are in fact the two sides of the same coin: both mechanisms could be involved simultaneously and differently depending on the visual display of the screen and the length of the text. Visual cues, informing about spatial relationships between parts of the text within a page, and sensorimotor cues furnished by the book handling and informing about spatial relationships between parts of the text disseminated among pages of the book, likely participate to the construction of the cognitive map of the text. In the present study, since we compared two books with visually identical pages, we focused more on the second aspect of reading.

Another aspect to consider which may help explain the poorer performance on reconstruction of chronology and temporality on a Kindle compared to paper, may be related to the “recursive dimension” of print (see e.g., Wolf, 2018). When reading lengthy texts, perhaps in particular narratives and novels, we occasionally need to backtrack to remind ourselves of, for instance, relations between characters, their names, or how events were interconnected. When we read in a print book, we can easily go back and check whenever needed, and we have immediate access to earlier pages whether they are five or fifty pages before the one page we’re currently reading. Obviously, we can also go back on a Kindle, but backtracking on a digital device is not as quick and effortless as with a paper book. Moreover, the reader’s task of locating information on earlier pages, spatially and temporally, is made more challenging with the lack of materiality of a digital text – whether on a Kindle or on an iPad. It may be that such a sense of added cognitive (and sensorimotor) effort discourages readers from going back to re-read earlier parts of a text when reading on a digital device, with a potential effect being a sub-optimal mental representation of spatiotemporal relations between events and/or characters. As this is the first experiment to compare the reading of a long, linear text on paper and screen, we recommend that future studies are designed to address this issue more specifically and in-depth. This could be done by, for instance, using text manipulations that can be assumed to trigger back-tracking and re-reading, for instance by systematically changing information in a way that will require updates in readers’ situation model (e.g., character names or goals; event locations; causal or temporal relationships between events). We may hardly conclude that reading comprehension was affected with e-book because most of the tests did not reveal differences between print and e-book. Yet, reading on an e-book seems to give rise to a less correct representation of the chronology of the events occurring in the story. Because temporal and causal links between events are usually closely connected, the understanding of the story might be somehow different in print and e-book. This point needs to be studied more precisely with longer texts and more specific measures.

Although steps were taken to ensure a more ecologically valid experimental setting than is often the case, it can be discussed whether the masking of page numbers (in both books) and also hiding the progress bar on the Kindle actually introduced an artifact that could somehow have influenced the results. Since we were primarily interested in assessing whether the difference in sensorimotor cues between a paper-based and a screen-based book made a difference for aspects of comprehension, we decided to strip both texts of any visual cues to text length. Based on the results of the present experiment, we can only conclude that sensorimotor cues play a role when reading a print book, whereas they are lacking when reading an e-book. The question remains whether visual cues, such as the progress bar on a Kindle, are equally efficient as sensorimotor cues. Therefore, future studies comparing long-form reading on paper and screen should include page numbers and/or other indicators of text localization, to assess whether such visual aids differently support mental reconstruction on paper and screen, as compared to sensorimotor cues. An additional limitation of the present study is that most of the participants were novices with respect to reading on a Kindle, and it can be claimed that they were not very avid readers of literature. To determine the role of medium expertise and preferences, and to empirically assess the assumptions underlying claims about so-called “digital natives,” future studies should compare reading different kinds of texts on an e-reader and on paper among expert Kindle (and similar device) readers. It would be interesting to also replicate this finding with participants who are more avid literary readers.

The stimulus in this experiment was a plot-based mystery story, to a large extent based on a chronological ordering of actions and events, so that the occurrence of an event in the story content – the “when in the story” – is often closely matched to the spatial location of the text passage in the book – the “where in the text.” While it is not implausible that similar results can be found by using other types of linear, chronologically structured texts (e.g., narratively presented historical accounts in textbooks), replications of the present study are needed, using different types and genres of texts (e.g., literary texts that are less plot-based; expository texts with low degree of narrativity). It may be that the ergonomic and visual affordances of different screen media may differently affect cognitive aspects of reading, depending on a number of variables relating to text (e.g., literary vs. non-literary; degree of narrativity; length; genre; structure/layout; complexity) as well as reader characteristics (e.g., medium/technology expertise and preference). The increasing popularity of the Bring-Your-Own-Device solution (see, e.g., Song, 2014) is testimony to the fact that for instance device *ownership* may be a significant factor in this equation.

Future research should also address the affective and emotional aspects of reading. Beyond applying an adapted version of Busselle and Bilandzic’s (2009) Narrative Engagement Scale, we did not include any measures of emotional and affective aspects. Given that the stimulus text is a mystery story by an established author, this may seem an unfortunate omission. Moreover, applied *post hoc*, rating scales are also liable to distortion and can more accurately be said to measure readers’ verbalized memory of what they may have felt at the time of reading (see e.g., Jacobs, 2016a,b). Ideally, offline measures of emotional aspects of reading should be complemented by online measures that are less prone to such distortions. Specifically, ratings and other verbal responses could be fruitfully complemented with online, indirect, behavioral measures such as eye tracking or electrodermal activity, in order to shed more light on the role of affective and emotional processes in perhaps especially long-form, literary reading. The development of sophisticated interdisciplinary and multi-methodological frameworks such as the Neurocognitive Poetics Model (Jacobs, 2015) is especially promising in this respect, applying a combination of measures at neural, behavioral and phenomenological levels in the study of literary – poetic as well as prose – textual material (see also Jacobs and Willems, 2018). Overall, we know too little about the ways in which digitization may affect emotional and motivational aspects of reading, and empirical research addressing such questions is much needed (see Kaakinen et al., 2018). As noted by Willems and Jacobs (2016), using literary texts as stimuli is, in this regard, a rich and largely untapped potential.

Limitations as the above notwithstanding, it seems safe to conclude that digitization brings with it the need to update existing models of reading in general, and of reading comprehension, in particular. Importantly, models should be elaborated and refined to account for the role of various features of media (e.g., print books, laptops, tablets, and e-readers) and their substrates (e.g., paper, electronic ink screens, LCD screens) on the reading of various types of texts, for different purposes. Mangen and van der Weel (2016) propose such an integrative, transdisciplinary model, accounting for the psychological, ergonomic, technological, social, cultural and evolutionary aspects of reading and how these are being affected by digitization. An exploratory model, it is intended to point to blanks in our knowledge of the differences between paper and screen reading, hence pointing out directions for future empirical research. The findings of the present experiment indicate that one salient textual parameter to pursue in future research comparing paper and screen reading, is text length and the ways in which a text may prompt re-reading, at various levels and for various reasons.

## Conclusion

Although it should be considered largely exploratory, the study adds to a growing body of evidence indicating that paper and screen reading may differ also in cases of linear, narrative reading where there are no hyperlinks to click on or multimedia content to process. Moreover, it illustrates the value of studying parameters not commonly addressed in reading research, such as haptic and tactile feedback. In the process toward more ecologically valid experiments in reading research, the study also contributes valuable insights into aspects of reading comprehension when the text is substantially longer than what is typical in empirical reading research of any disciplinary orientation.

## Author Contributions

AM and J-LV conceived and designed the experiments. GO and J-LV performed the experiments. J-LV analyzed the data. AM and J-LV wrote the manuscript.

## Conflict of Interest Statement

The authors declare that the research was conducted in the absence of any commercial or financial relationships that could be construed as a potential conflict of interest.

## Footnotes

1 See, for instance, Klatzky and Lederman (1988), Lederman and Klatzky (1998), and Klatzky and Lederman (2002) for more in-depth exploration of these closely related phenomena.

2 Cronbach’s alpha for the original Narrative Engagement Scale was 0.80 (see Table 3 in Busselle and Bilandzic, 2009).

3 Kendall tau distance is equivalent to the number of swaps required to place one list in the same order as the other list. If both classifications are identical, the Kendall tau distance = 0; if both classifications are totally in opposite, the Kendall tau distance = N (N-1) / 2 (in this case *N* = 14), resulting in a maximum distance of 91. The intermediate arrangements have a distance from the correct plot arrangement ranging from 0 to 91.
